# Benefits of rescreening newborns of mothers affected by autoimmune hypothyroidism

**DOI:** 10.1530/ETJ-22-0060

**Published:** 2022-07-17

**Authors:** Paolo Cavarzere, Laura Palma, Lara Nicolussi Principe, Monica Vincenzi, Silvana Lauriola, Rossella Gaudino, Virginia Murri, Luigi Lubrano, Giuliana Rossi, Alessia Sallemi, Ermanna Fattori, Marta Camilot, Franco Antoniazzi

**Affiliations:** 1 Pediatric Division, Department of Pediatrics, University Hospital of Verona, Verona, Italy; 2 Pediatric Section, Department Surgical Sciences, Dentistry, Gynecology and Pediatrics, University of Verona, Verona, Italy; 3 Regional Center for Newborn Screening, Diagnosis and Treatment of Congenital Metabolic and Endocrinological Diseases, Verona, Italy; 4 Neonatal Intensive Cure Unit, Department of Pediatrics, University Hospital of Verona, Verona, Italy; 5 Pediatric Division, Hospital of San Bonifacio, Verona, Italy; 6 Pediatric Division, Hospital of Legnago, Verona, Italy; 7 Pediatric Division, Hospital of Mestre, Venezia, Italy; 8 Pediatric Division, Hospital of Venezia, Venezia, Italy; 9 Pediatric Division, Hospital of Negrar, Verona, Italy; 10 Regional Center for the Diagnosis and Treatment of Children and Adolescents Rare Skeletal Disorders, Pediatric Clinic, Department of Surgical Sciences, Dentistry, Gynecology and Pediatrics, University of Verona, Verona, Italy

**Keywords:** maternal autoimmune hypothyroidism, neonatal hypothyroidism, newborn screening, anti-thyroid antibodies

## Abstract

**Introduction:**

Infants of mothers with autoimmune hypothyroidism (AH) are at risk of developing late-onset hypothyroidism, often escaping at newborn screening. This condition might be caused both by the action of maternal antibodies and/or by maternal treatment.

**Objectives:**

The aim of this study is to evaluate the prevalence of AH in the mothers of children born in Veneto region, Italy, and to define what is the most appropriate management for these newborns.

**Methods:**

Newborns of six different hospitals with a mother suffering from AH and with negative neonatal screening for congenital hypothyroidism (CH) were included in the study. Between 15 and 20 days of life, we collected a serum sample for the evaluation of thyroid function (thyroid-stimulating hormone (TSH), free thyroxine (FT4), free triiodothyronine (FT3)) and anti-thyroid antibodies. On the same occasion, a capillary blood sampling was performed for a second screening test.

**Results:**

Maternal AH has a prevalence of 3.5%. A total of 291 newborns were enrolled from November 2019 to May 2021. Whereas the 11.4% of infants had a slight elevated serum TSH (>6 mU/L) and required a follow-up, only 2 children presented an elevated TSH level at the second screening test. One of these, with the gland *in situ*, showed persistently elevated serum TSH levels and required treatment with levothyroxine.

**Conclusions:**

Maternal AH rarely caused neonatal thyroid dysfunction. We suggest to reassess newborns from mothers with AH 15 days after birth by means of a second neonatal screening test. This procedure avoids false negatives due to maternal thyroid status, is less invasive and cheaper than the serum TSH evaluation, and prevents a long follow-up.

## Introduction

During pregnancy, normal thyroid function is essential to ensure the regular development of the fetus (1, 2). Overt or subclinical maternal hypothyroidism can modify, in fact, the course of pregnancy and the development of the fetus (3, 4). The incidence of gestational hypothyroidism widely varies from 0.14 to 11.1% in different studies (5, 6, 7). During pregnancy, the most frequent cause of maternal hypothyroidism is autoimmunity, defined by the presence of maternal anti-thyroid antibodies against thyroid peroxidase (TPOAb), thyroglobulin (TGAb), and/or thyrotropin receptor (TRAb). Its overall prevalence is 7.8%, ranging from 5 to 20% in different studies (5). TPOAbs cross the placenta, but it is not currently clear whether they interfere with the function of the fetus’ thyroid (8).

In children from mother with autoimmune hypothyroidism (AH), an increased prevalence of congenital hypothyroidism (CH) is described (1, 9); however, no study has ever correlated these data with the positivity for TPOAb, rather this occurrence was caused by TRAbs with inhibiting effect (9, 10). This condition seems to be extremely rare, occurring from 1:84,700 to 1:310,000 (11, 12).

On the basis of these considerations, many issues remain to be addressed: are newborns with a mother with AH at the risk of neonatal CH? Should these neonates be managed with particular attention? Is maternal therapy with levothyroxine protective for the newborns? Could maternal treatment lead to a false negative newborn screening result for CH, delaying the necessary replacement therapy? In particular, to avoid the latest, some authors have suggested to perform serum thyroid function tests between 15 and 30 days of life in all children with mothers with AH (13, 14). In 2010, Rovelli et al. demonstrated that approximately 28% of these newborns with blood tests performed between the third and fourth day and later on at 15–30 day had a moderate increase in serum TSH, although the screening at birth was negative for all of them. After 1 month, 93.3% of these showed a normalization of TSH values and only 2.2% of cases required replacement treatment, stopped within the second year of life (12).

On the contrary, in 2016, McGovern et al. claimed that neonatal screening performed between the 3rd and 5th day of life in newborns with mothers affected by AH proved effective in identifying all cases of hypothyroidism, without the need for further confirmatory blood tests (15).

Despite the absence of common guidelines for the management of these newborns (16), since 2010, following Rovelli’s suggestion, our screening center has been recommending to perform thyroid function test on blood at about 15–20 days of life in all newborns born to a mother with AH. Upon an abnormal result, patients are sent to pediatric endocrinology to carry out further investigation and, if necessary, start a replacement therapy.

Our study aims to analyze thyroid function both on serum and on newborn screening in term neonates of mothers with AH and to evaluate the prevalence of AH during gestation in Veneto, a North Eastern Italian region. On the basis of the collected data, we intend to pinpoint what is the most appropriate neonatal management for these newborns.

## Patients and methods

### Patients

We enrolled all newborns of mothers affected by AH, born from November 2019 to May 2021 in six different hospitals of Veneto region, Italy.

Inclusion criteria were as follows: negative newborn screening for CH, maternal AH confirmed by presence of anti-thyroid antibodies (TPOAb and/or TGAb), gestational age (GA) ≥ 37 weeks, and birth weight > 2500 g. Exclusion criteria were as follows: GA < 37 weeks, birth weight < 2500 g, presence of congenital disorders, chromosomal abnormality, or other causes of admission to intensive care unit.

Of each child, we collected GA, TSH level at newborn screening test, dose of levothyroxine taken by the mother at the delivery, and birth auxological data: weight, length, and head circumference.

Between 15th and 20th day of life, blood determinations of TSH, FT4, FT3, TPOAb, TGAb, and TRAb were performed in all enrolled.

In the same time, we evaluated TSH level on a capillary blood sample collected from the newborn heel, dried on filter paper, and sent to screening laboratory, as a second screening test.

To standardize the serum levels performed in different laboratories, we calculated the z-score.

According to the more recent consensus guidelines (17, 18), we considered thyroid function as normal in the newborn when FT4 was in the reference range in the presence of a TSH level below 6 mU/L; in these cases, no further tests were required. When FT4 was in the reference range and TSH level was between 6 and 20 mU/L, a clinical and laboratory follow-up was suggested. Finally, if TSH level was higher than 20 mU/L, regardless of FT4 value, or if FT4 was lower than the reference range, CH was diagnosed and a replacement therapy with L-T4 begun.

To reduce inaccuracies in the data collection caused by the different birth centers, the data were reviewed by the same investigator and an attempt was made to eliminate any selection biases.

### Methods

All newborns were submitted to neonatal screening to identify CH through the heel prick blood samples collected at 48-72 h after birth, dried on filter paper, and sent to our screening laboratory in 24 h. In 2016, a national prescription recommended the blood collection for all newborn screening programs between 48 and 72 h of life. In fact, concomitantly with the newborn screening for CH, other screening tests are performed in our center in order to investigate different metabolic disorders and congenital adrenal hyperplasia.

The screening program is based only on the TSH measurement in dried blood spot. When the TSH value was above the retest value (9 mU/L blood in our screening center), the analysis was repeated in double on the same blood sample. If TSH value (in two out of three determinations) exceeded the cut-off of 10.5 mU/L blood, the newborn was recalled and thyroid function tests on serum were performed.

The test was performed using GSP Neonatal hTSH kit on GSP platform (Perkin Elmer Wallac Oy, Turku, Finland). It is based on a direct sandwich technique; in this way, two monoclonal antibodies recognize separate antigenic determinants on the hTSH molecule. The maximum coefficients of variation intra- and inter-assay are 7 and 8.7%, respectively.

Serum values of TSH, FT4, FT3, TPOAb, and TGAb were evaluated using an immunological technique based on electro-chemiluminescence in two sites on a solid surface (Elecsys, Roche Diagnostics GmbH, Mannheim, Germany). The coefficients of variation of intra-assay and inter-assay are 4.3 and 7.1%, respectively. The quantitative assay of TRAb is evaluated by an automatic immunofluorescence technique (BRAHMS TRAK human KRYPTOR, Thermo Scientific, Hanningdorf, Germany). The coefficients of variation of intra- and inter-assay are 4.3 and 7.1%, respectively.

### Ethical issues

The study was conducted in line with Helsinki Declaration II and approved by the ethical committee for clinical trials of Verona and Rovigo (Prog. 2585CESC). Written informed consent was obtained from the parents of each patient.

### Statistical analysis

The statistical analysis was conducted using SPSS 25 for Windows. Normal distribution was assessed by the Kolmogorov–Smirnov test. Comparisons between groups were performed using Student’s *t*-test or the Mann–Whitney U test, whenever appropriate. Rates were compared by χ^2^ or Fisher’s exact test, whenever appropriate. The correlations were evaluated using the Pearson or Spearman tests, as appropriate. Data are expressed as frequency, median plus range, or mean ± s.d., as appropriate. Statistical significance was reached as *P* - values < 0.05, and all tests were two-sided.

## Results

From November 2019 to May 2021, 291 newborns (51.9% females, 48.1% males) born to mothers affected by AH were included in this study. During the same period, we identified CH in a female newborn with mother affected by AH. Since 8386 healthy term babies were born in this period in the hospitals that took part in this study, the prevalence of newborns to mothers with AH in our region is estimated to be 3.5%, without statistically significant difference among the different hospitals involved.

**Figure 1:**
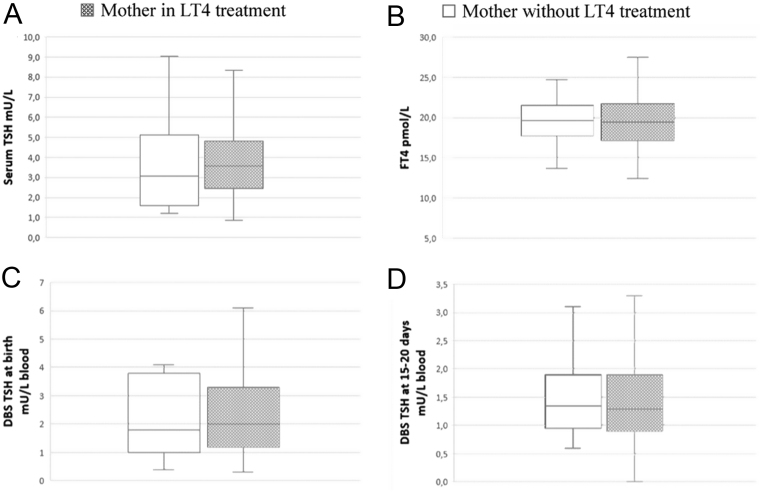
Serum levels of TSH (A), FT4 (B), dried blood spot TSH at birth (C), and dried blood spot TSH at 15−20 days of life (D) in patients divided in relation to maternal levothyroxine treatment during pregnancy. The median values are represented as a horizontal line. The edges represent respectively the 25th and the 75th centile of the cohort. Vertical lines represent the range. FT4, free thyroxine; LT4, levothyroxine; DBS, dried blood spot; TSH, thyroid stimulating hormone.

All the mothers were in condition of euthyroidism during gestation. The majority of them (74.2%) assumed chronic levothyroxine with a mean dose of 72 µg/day, 4.1% did not follow any therapy, whereas there was no information available on the remaining 21.7%. No significant correlations were detected between mother’s therapy and TSH at neonatal screening or thyroid function at 15–20 days of life (Fig. 1).

**Figure 2:**
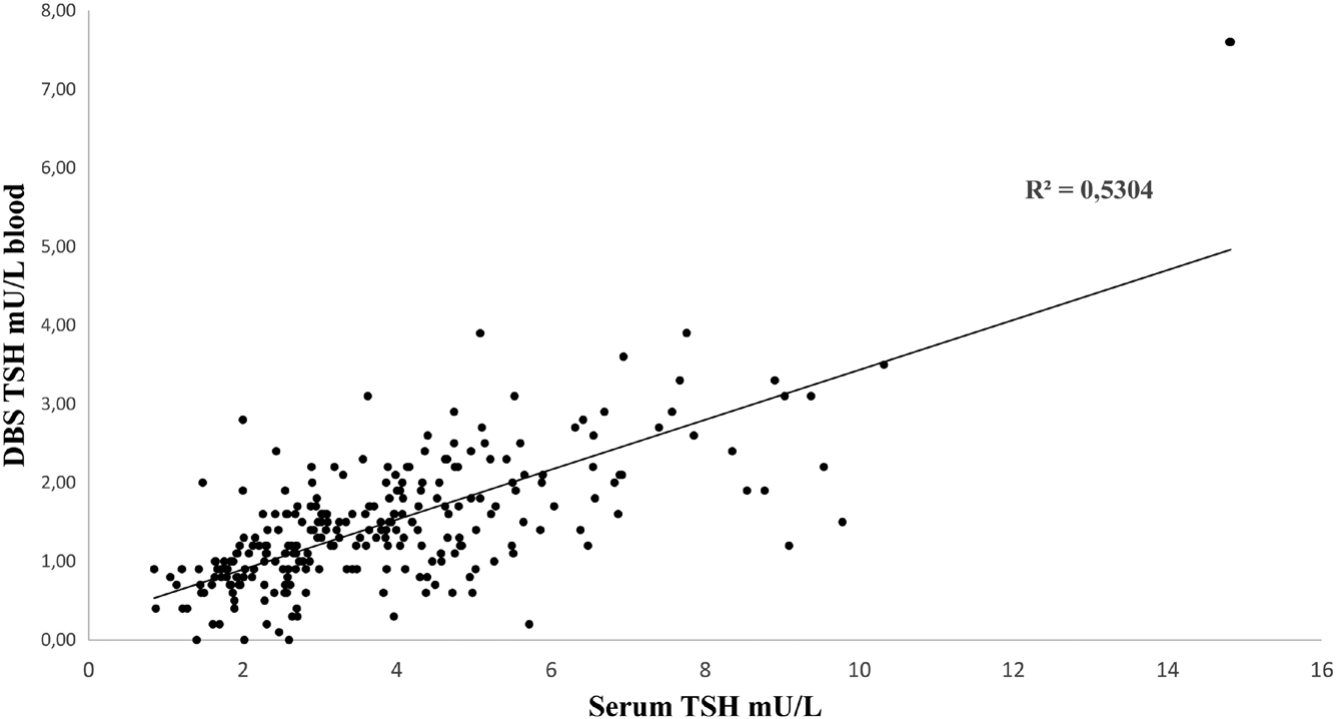
Correlation between serum TSH values and dried blood spot TSH at 15–20 days of life (R^2^= 0.5304, *P*  < 0.01). DBS, dried blood spot.

The majority of newborns (72%) was born from spontaneous childbirth, the remaining from cesarean delivery. Auxological data at birth of all newborns are reported in Table 1. All babies enrolled in the study had a negative neonatal screening test for CH at birth, with a TSH value of 2.60 ± 1.70 mU/L blood. Thyroid function on serum was performed at 18.5 ± 3.7 days of life; at the same time, a second screening test was repeated on a heel prick withdrawal. The serum and screening TSH were 3.81 ± 1.98 (0.85–14.82) mU/L and 1.46 ± 0.83 (0.0–7.63) mU/L blood, respectively, with a significant correlation between these two values (R^2^ = 0.5304; *P*  < 0.01, Fig. 2). At the second screening test, only two children presented an elevated TSH level on dried blood spot: a male baby that had a value of 206 mU/L blood and another male with a value of 7.6 mU/L blood. In the former case, serum TSH confirmed the screening result, with a value of 356 mU/L, the FT4 was 4.37 pmol/L, consequently a treatment with levothyroxine was immediately started. He had a gland *in situ* and did not present anti-thyroid antibodies. His mother regularly consumed levothyroxine supplementation. The latter baby presented a serum TSH of 14.8 mU/L with normal FT4 level; he was followed over time, up to the total normalization of thyroid hormone at the 24th day of life without therapy.

**Table 1:** Auxological data at birth of the enrolled newborns.

	Male (*n* = 140)	Female (*n* = 151)	Tot (*n* = 291)
Gestational age (weeks)	39.4 ± 1.2	39.2 ± 1.1	39.3 ± 1.2
Weight (g)	3487.9 ± 427.4	3330.0 ± 361.6	3406.2 ± 401.9
Length (cm)	48.7 ± 6.0	49.0 ± 5.0	48.8 ± 5.5
Head circumference (cm)	37.5 ± 6.6	36.1 ± 5.8	36.8 ± 6.3

The 11.4% of infants had a serum TSH value higher than 6 mU/L (7.99 ± 1.76 mU/L vs 3.42 ± 2.15 mU/L blood on dried blood spot at 15 days of life), which required a clinical and laboratory follow-up, according to ESPE guidelines. All of them showed a normalization of TSH values within 4 months of life (65.5 ± 56.0 days of life). No infants, with the exception of the abovementioned treated baby, presented low FT3 and/or FT4 levels. Of the infants, 32.7% presented elevated FT4 levels, the 69% of their mothers assumed levothyroxine treatment, but no significant correlation between FT4 levels and the dose of maternal l-thyroxine was detected. Interestingly, we found a negative relationship between the FT4 level and the days of life in which the sample was taken (r = –0.21, *P*  < 0.001).

**Figure 3:**
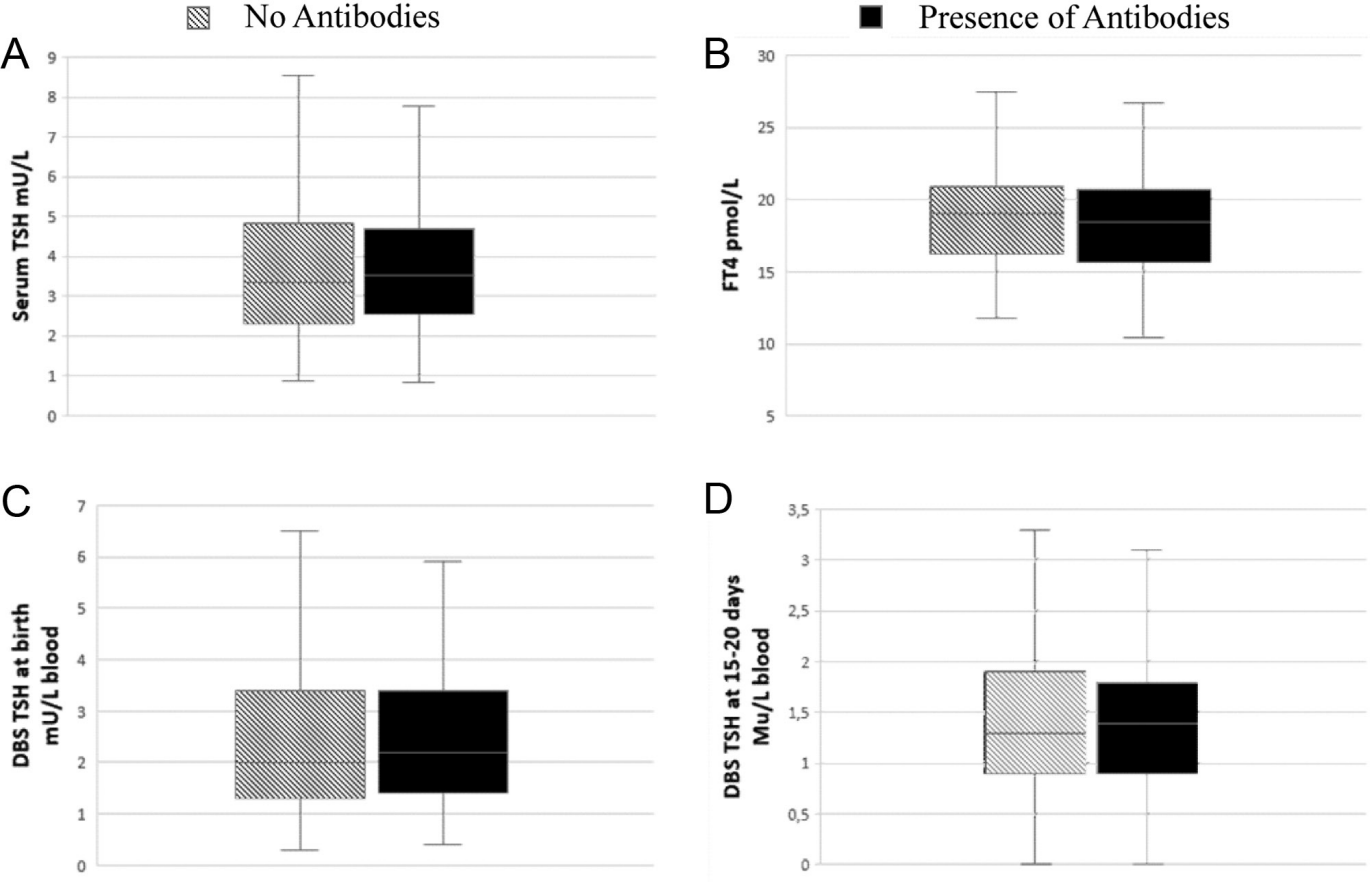
Serum levels of TSH (A), FT4 (B), dried blood spot TSH at birth (C), and dried blood spot TSH at 15–20 days of life (D) in patients divided according to antibody status. The median values are represented as a line. The edges represent respectively the 25th and the 75th centile of the cohort. Vertical lines represent the range. DBS, dried blood spot; FT4, free thyroxine; TSH, thyroid stimulationg hormone.

Of the babies, 41.7% showed at least one of the anti-thyroid antibodies: in particular, 37.9% showed TPOAb and 9.7% TGAb. On the contrary, TRAbs were negative in all children. No significant correlation was detected between antibody status and TSH at neonatal screening, not even with thyroid function at 15–20 days of life (Fig. 3). Babies with serum TSH greater than 6 mU/L did not show an increase in antibody titer relative to the others.

## Discussion

To our knowledge, this is the first prospective study that evaluates the need to monitor thyroid function in newborns born to mothers with AH, a not rare condition. On the basis of our results, we suggest to submit all these newborns for a second screening test at 15th day of life, avoiding a longer and more expensive follow-up.

In our area, the prevalence of maternal AH (3.5%) is lower than that reported in the literature, in which a prevalence of 7.87% was evidenced (5). However, the population covered by this study was represented only by children born at term, with a birth weight higher than 2500 g, not suffering from chromosomopathies and not admitted to neonatal intensive care unit, while the data reported in the literature is cumulative for the entire population including premature births. An association between hypothyroidism during pregnancy and an increased risk of preterm birth or of low birth weight newborns is known (4, 19). Consequently, these differences could explain the discrepancy with previous data. Furthermore, other studies reported a prevalence of hypothyroidism in women of childbearing age ranging between 2.5 and 4.8%, identifying AH as the main cause (6, 7, 20, 21), in line with our prevalence.

Newborn babies of mothers with this thyroid disorder must be considered a category of children worthy of attention because they are at risk of developing a delayed hypothyroidism, not recognizable at newborn screening performed after birth. This risk is essentially the consequence of two factors: the presence of antibodies inhibiting thyroid function derived from the mother and can persist for several months (1, 22), and the maternal treatment with L-T4 which could mask an already present hypothyroidism (11). The role of maternal antibodies in the genesis of this type of hypothyroidism is still a matter of debate. In fact, it seems that maternal anti TPOAbs do not affect the thyroid function of the fetus and newborn (8). On the contrary, in a variable percentage between 10 and 20%, the anti-TRAbs of pregnant women with AH have an inhibiting effect. These antibodies can pass the placenta and inhibit fetal production of thyroid hormones causing transient or permanent neonatal hypothyroidism (22), the incidence of which appears to be very low, ranging between 1 in 40,000 births and 1 in 570,000 births; in the most severe cases, this form can be recognized at newborn screening (11, 12, 13). Our data confirm this situation since the presence of high anti-TPOAb titers did not result as a risk factor for neonatal hypothyroidism and the affected baby of our cohort did not present anti-TPOAb. We did not find significant difference between the hormone levels of patients with or without TPOAb. Furthermore, none of our patients presented positive TRAb nor did the patient with overt hypothyroidism. However, it should be pointed out that the common kits used for the assay of anti-TRAb do not have sufficient sensitivity to distinguish the presence of inhibiting antibodies from stimulating ones; therefore, this data is difficult to interpret.

The role of maternal treatment on the thyroid function of the newborn is not well defined (11). No correlation was found between the serum levels of FT4, FT3, TSH, blood spot TSH, and the dose of L-T4 taken by the mother. Furthermore, it seems that the day in which the withdrawal was carried out influenced the levels of thyroid hormones (23). An inverse correlation was found between the levels of FT4 and the day of sample collection. This data can be explained by the physiology of the release of the thyroid hormones. It is known that after birth there is an increase in the level of thyroid hormones followed by a progressive decrease to normal levels over 3–5 weeks (24). We can therefore conclude that an elevated FT4 level does not depend only on maternal treatment but, rather, on the physiological trend of hormonal levels in the neonatal period. Accordingly, some of our patients identified with elevated FT4 were actually children of untreated mothers.

The main issue remains whether a newborn of a mother with AH should undergo thyroid function tests and if the tests should be performed only if the mother is treated with L-T4 or in all newborns. Considering the harmlessness of anti-TPOAb to fetal and neonatal thyroid function, the rarity of transient neonatal hypothyroidism induced by anti-TRAb with inhibitory significance and the likely recognition of this condition at neonatal screening, the answer would be ‘no’ (1). Many authors agree with this approach by pointing out precisely the rarity of the problem, the practical and organizational difficulties of carrying out a blood sample from all these children after dismissal from nursery, as well as the concern that this practice would cause to parents (8, 25). Similarly, an Irish study highlights that in 1 year, no child of mother with AH presented a delayed TSH increase, considering the newborn screening performed in the first days of life to be sufficient (15). However, in Ireland, newborn screening is performed at later age, between 72 and 120 h of age, likely reducing the influence of the high doses of L-T4 needed in pregnancy. Finally, more recently some authors declared that no added clinical benefit was found in retesting newborns of hypothyroid mothers in addition to the newborn screening program (26).

On the contrary, other studies deem useful a reassessment of the newborn of mothers with AH (13, 14). Our identification of hypothyroidism in a newborn born by a mother with AH in levothyroxine supplementation, with a negative neonatal screening for CH shows the importance of a control at 15 days of life in these newborns. In this case, the absence of adequate follow-up would in fact have hindered the identification of the disease, provoking important neurological consequences (27). In addition, maternal levothyroxine treatment might affect newborns’ TSH levels and consequently their screening results. In our view, to avoid neurological sequelae even in a single child is a priceless result, not to mention that the cumulative economic benefits, which might derive from preventing intellectual disability, far outweigh the direct and indirect costs of screening, treatment, and surveillance throughout the life of the affected child (28, 29). Moreover, given the relevant prevalence of maternal AH and the limited number of cases enrolled in the present study, the number of hypothyroid newborns escaping to the screening at birth might be remarkable. Therefore, we retain that it is preferable to repeat a second dried blood spot at 15 days of life rather than to submit all these newborns to venous withdrawals with the risk of a prolonged and often unnecessary follow-up. All newborns were in fact negative at the screening repeated at 15–20 days of life, with the exception of the abovementioned child. The practice of collecting a dried blood spot for TSH determination at 15–20 days of life for all newborns with a mother affected by AH allows avoiding unnecessary and expensive blood exam, source of anxiety for parents and stress for the babies.

The percentage of newborns with increased serum TSH levels is slightly higher than what previous reported in an Italian study of 2010 (14), according to which only 6.7% of newborns showed an increase in TSH at 15 days of life. However, in that study, the 2.2% of patients required L-T4 therapy due to persistent hyperthyrotropinemia during follow-up. On the contrary, none of infants included in our study required therapy, except for the affected child. All children in which TSH levels were above the reference range showed normalization of TSH at subsequent controls. For this reason, it did not seem necessary to perform further tests, in particular the thyroid ultrasound analysis which, according to Rovelli et al., could instead be recommended in case of persistent hyperthyrotropinemia (14). As further confirmation of the usefulness of a second screening performed at 15–20 days of life, we highlighted a very strong positive correlation between the TSH value found on second dried blood spot and the corresponding level of serum TSH. Furthermore, a heel prick blood withdrawal is less invasive, practically painless for the newborns, and cheaper than venous sampling. Screening procedure is significantly less expensive than blood determination of TSH, FT3, and FT4. This analysis in fact takes into consideration two different types of costs: for the National Health System and for the patient. Analyzing the costs of the National Health System, we compared the costs of screening test versus the costs of a venous sample: in our center, the total cost of a second screening amounts to about 4 euro versus the cost of 10 euro for each serum analyte. Therefore, leaving out the dosage of antibodies, which are indicated only if TSH was altered, and considering only the levels of TSH and FT4, the cost of venous sample is five times greater. Moreover, it is worth considering that while the execution of a second card routinely proceeds through the hospital system, the execution of the serum assay falls on the parents, and although recommended, some parents might postpone or try to avoid the execution of the blood test, with the concrete possibility that some newborns arrive at the diagnosis of CH too late. Finally, serum sample results a major source of anxiety for parents.

This proposal should certainly be verified through specific studies with higher numbers of patients, since at the moment maternal family history for AH is not foreseen among the categories for which the repetition of screening is indicated such as preterm, low weight, twins (17, 18, 27).

Besides the limited number of mother/newborn couples enrolled, the main limitation of our study is the lack of a control population. Another limitation is that we have only considered the term children of mothers with AH with negative screening. This is due to the fact that they are the children who risk being lost at screening, as premature babies are routinely retested at 15 and 30 days of age, whereas full-term babies with positive screening are automatically recalled.

In conclusion, AH in pregnancy is not a rare condition and is detected in 3.5% of women. It may cause a mostly transient neonatal thyroid dysfunction and sometimes late CH onset. Therefore, we suggest to reassess newborns from mothers with AH 15 days after birth through a second neonatal screening, which have resulted to be less invasive and cheaper than the serum TSH evaluation and which allows to avoid a long and useless follow-up, often source of anxiety for parents.

## Declaration of interest

The authors declare that there is no conflict of interest that could be perceived as prejudicing the impartiality of the research reported.

## Funding

This work did not receive any specific grant from any funding agency in the public, commercial, or not-for-profit sector.
